# Otoacariasis in East China: a case series of three tick bites with diagnostic and management challenges

**DOI:** 10.3389/fpubh.2026.1946165

**Published:** 2026-10-06

**Authors:** Yongzhi Niu, Wenxiu Jiang, Kaixuan Chen, Fei Che, Xiaojun Wang, Wenhuan Wang, Xiaoting Wang, Wenhui Pang

**Affiliations:** 1 Department of Otolaryngology-Head and Neck Surgery, The Affiliated Hospital of Qingdao University, Qingdao, Shandong, China; 2 Department of Otolaryngology, Eye and ENT Hospital, Fudan University, Shanghai, China; 3 Department of Clinical Laboratory, The Affiliated Hospital of Qingdao University, Qingdao, Shandong, China; 4 Department of Otolaryngology-Head and Neck Surgery, Shandong Provincial Hospital Affiliated to Shandong First Medical University, Jinan, Shandong, China

**Keywords:** case series, diagnostic challenge, external auditory canal, one health, otoacariasis, tick bites

## Abstract

**Objective:**

Tick bites in the external auditory canal are rare but can pose diagnostic and management challenges in primary care settings. This case series aims to highlight diagnostic challenges and public health implications of otoacariasis in East China.

**Methods:**

We retrospectively reviewed three cases of tick bites that initially posed diagnostic and management challenges at primary hospitals in Qingdao, Jinan, and Shanghai. Clinical data, including presentation, initial diagnostic assessment, treatment, and follow-up, were extracted from electronic medical records.

**Results:**

Case 1: a 70-year-old female with an engorged tick abdomen mistaken for the tympanic membrane. Case 2: a 3-year-old girl with retained tick mouthparts after forceful removal. Case 3: a 5-year-old girl with a tick on the tympanic membrane misidentified as a common insect. Key diagnostic clues included tick morphology, dark red feces, and leg movement. Topical 2% lidocaine effectively immobilized ticks for atraumatic extraction.

**Conclusion:**

These cases highlight a diagnostic and management gap in primary care settings in East China, underscoring the need for enhanced physician education and public awareness as a key public health strategy to promote early recognition and improve patient outcomes in the region.

## Introduction

Tick bites are occasionally encountered in clinical practice and may lead to potentially fatal diseases, including severe fever with thrombocytopenia syndrome (SFTS) and Lyme disease (1). Tick bites are primarily observed on the skin of the head, neck, arms, and legs in humans, but they are rarely found in the ear. When this occurs, they can lead to serious otologic complications, including vertigo, tinnitus, hearing loss, and even facial paralysis (2–6). Accordingly, accurate identification and prompt removal of the tick are critical to mitigate tick-borne diseases and co-infection (7). If a tick bite is mistaken for the bite of another organism, aggressive removal may occur leading to serious complications. Therefore, identifying tick bites in the ear presents a significant clinical challenge for otorhinolaryngologists. Beyond the clinical challenge, this diagnostic difficulty represents a public health concern, as misdiagnosis can lead to inappropriate management, increased morbidity, and missed opportunities for preventing tick-borne diseases at the community level.

Here, we describe three cases of tick bites involving the ear that initially posed diagnostic and management challenges at primary hospitals in Qingdao, Jinan, and Shanghai (East China). We focus on the clinical presentation, diagnostic pitfalls, and key otoscopic features—including tick morphology, dark red feces, and leg movement—that facilitate accurate identification and prompt removal, aiming to provide guidance for otorhinolaryngologists and raise awareness of otoacariasis in the East China region.

## Results

### Case 1: diagnostic challenge of tympanic membrane penetration by tick in the ear

On July 18, 2021, a 70-year-old woman presented to the otolaryngology emergency department of a tertiary hospital in Qingdao (Shandong Province, East China), with a 2-week history of intermittent tinnitus, aural fullness, and pruritus in the right ear. She had no history of ear disease or pet ownership but resided in a rural area, with associated environmental exposure. She had initially presented to a primary hospital, where she was told that her eardrum had been perforated, possibly by a parasite. At our institution, endoscopy revealed a milky-white cystic mass filling the external auditory canal and obscuring the tympanic membrane. The abdomen of the tick had been mistaken for the tympanic membrane, and one leg for a living organism (Figure 1A). Dark red feces were observed in the external auditory canal (Figure 1B). Post-removal endoscopy later revealed an intact tympanic membrane (Figure 1C). The active, engorged tick was visualized after removal (Figure 1D). A video recording documenting tick leg movement is available as Supplementary Video S1.

**Figure 1:**
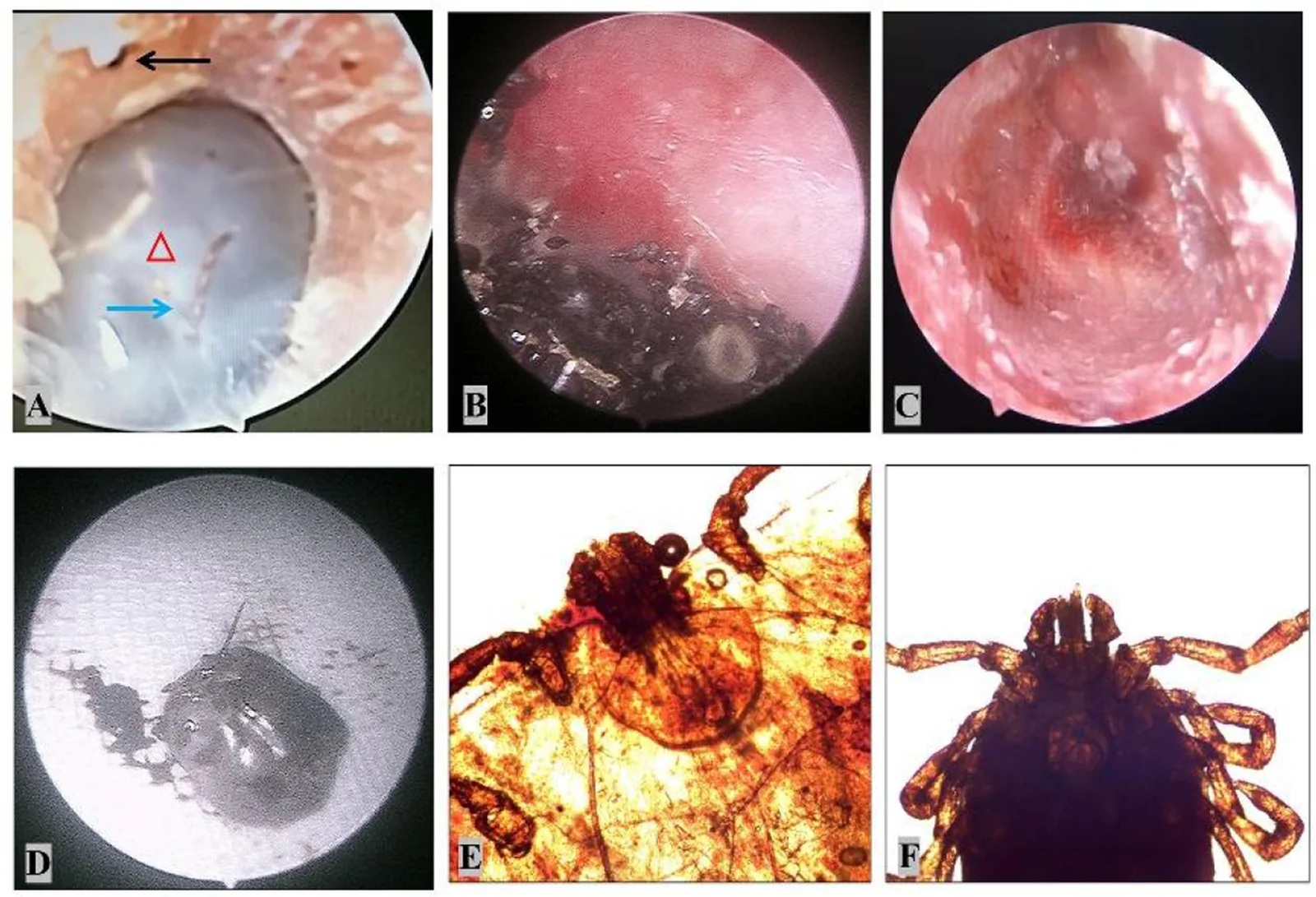
**(A)** Electro-otoscopic view of the right ear. The red triangle indicates the engorged tick abdomen (mistaken for the tympanic membrane); the blue arrow points to a tick leg; the black arrow indicates dark red feces in the external auditory canal. **(B)** Close-up of tick feces in the external auditory canal. **(C)** Post-removal electro-otoscopic view showing the intact tympanic membrane. **(D)** Electro-otoscopic view of the engorged tick after removal. **(E)** Microscopic view of the tick mouthparts, confirming complete removal without residual fragments. **(F)** Microscopic view of the extracted tick, showing the scutum (dorsal shield), four pairs of legs, and capitulum (mouthparts), confirming family Ixodidae.

The tick was promptly removed in the outpatient setting without general anesthesia. After instilling 2% lidocaine into the external auditory canal for 15 min, the solution was aspirated, and the immobilized tick was extracted using microforceps. The canal was irrigated with normal saline to remove residual feces. Slight erythema at the bite site indicated spontaneous detachment after feeding. The intact specimen was examined under a stereomicroscope by entomology specialists from the dermatology department. Mouthparts were intact, confirming complete removal without residual fragments (Figure 1E). The specimen was confirmed as an engorged hard tick (family Ixodidae) based on body size, scutum, capitulum, and leg segmentation (Figure 1F). Species identification was cross-referenced with regional taxonomic databases, consistent with peak seasonal activity in northern China (July). Routine blood tests were normal. Prophylactic oral minocycline (200 mg/day) and levofloxacin ear drops were prescribed for 7 days. At 1-month follow-up, the patient remained asymptomatic with normal vital signs and laboratory findings.

### Case 2: retained tick mouthparts in the helix of a child

On July 1, 2024, a 3-year-old girl was admitted to our otolaryngology department of a tertiary hospital in Jinan (Shandong Province, East China), with a one-day history of a foreign body in the right ear. The previous day, she had traveled with her parents to a grassland area on the outskirts of Jinan, where a tick-like organism was observed attached to the right crus of the helix. At a local hospital, the organism was forcibly extracted, leaving a black, dot-like foreign body at the bite site with surrounding redness, swelling, and tenderness (Figure 2A). No otorrhea, tinnitus, or hearing loss was reported. A tick bite was suspected.

**Figure 2:**
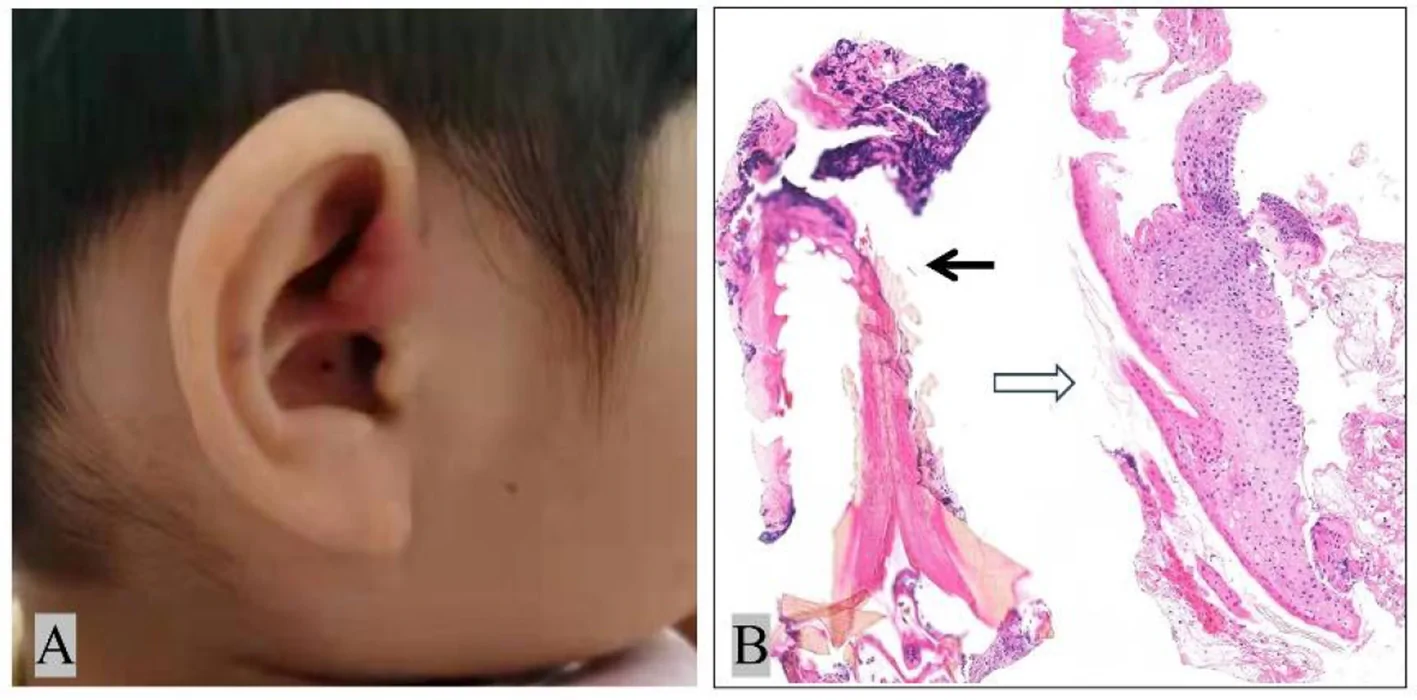
**(A)** Redness, swelling, and tenderness observed in the right crus of the helix following tick removal. **(B)** Pathological examination revealing foreign bodies with burr-like and serrated edges. Black arrow indicates retained tick mouthparts; white arrow indicates auricular cartilage and overlying skin.

Upon referral to our department, an infectious disease specialist confirmed the diagnosis. The child underwent surgical exploration under general anesthesia. A fusiform incision was made in the swollen helix crus, and the retained foreign body was completely excised. Pathological examination revealed burr-like, serrated structures consistent with tick mouthparts (hypostome and chelicerae), confirming retained mouthparts from an ixodid tick (Figure 2B). Although the tick body was missing, the morphology allowed for species identification as a probable *Haemaphysalis* or *Dermacentor* species, both prevalent in East China and known to inhabit grassland environments. Routine blood tests showed leukocytosis, suggesting local infection. Levofloxacin ear drops were prescribed; Minocycline was withheld due to the patient's age (risk of tooth discoloration in children under 8 years*)*. At 6-month follow-up, the child was in good health.

### Case 3: tick attached to the tympanic membrane misdiagnosed as a common insect

On September 1, 2025, a 5-year-old girl presented to the otolaryngology clinic of a tertiary hospital in Shanghai (East China) with a 2-day history of right otalgia, pruritus, and a sensation of movement in the ear. She had been playing in a grassy suburban park in the Pudong New Area of Shanghai the previous day. At a local community hospital, otoscopy revealed a small, dark-brown organism adherent to the tympanic membrane; it was dismissed as a common insect, and attempted suction removal failed.

At our institution, examination under an otologic microscope revealed a dark-brown organism firmly attached to the wall of the external auditory canal. Under low magnification, the tick appeared small and slender (Figure 3A), which likely contributed to the initial misidentification; however, high-magnification examination revealed a fully sclerotized scutum and robust legs characteristic of an adult hard tick (Figure 3B). The tick was alive, with slow leg movement. After instilling 2% lidocaine into the external auditory canal for 10 min, the immobilized tick was carefully extracted using microforceps. The intact specimen was confirmed as an adult hard tick (family Ixodidae) with intact mouthparts. The parents were counseled on monitoring for tick-borne diseases. Prophylactic antibiotics were withheld due to complete removal and the patient's age. At 1-month follow-up, the child remained asymptomatic, and otoscopy revealed a healed tympanic membrane.

**Figure 3:**
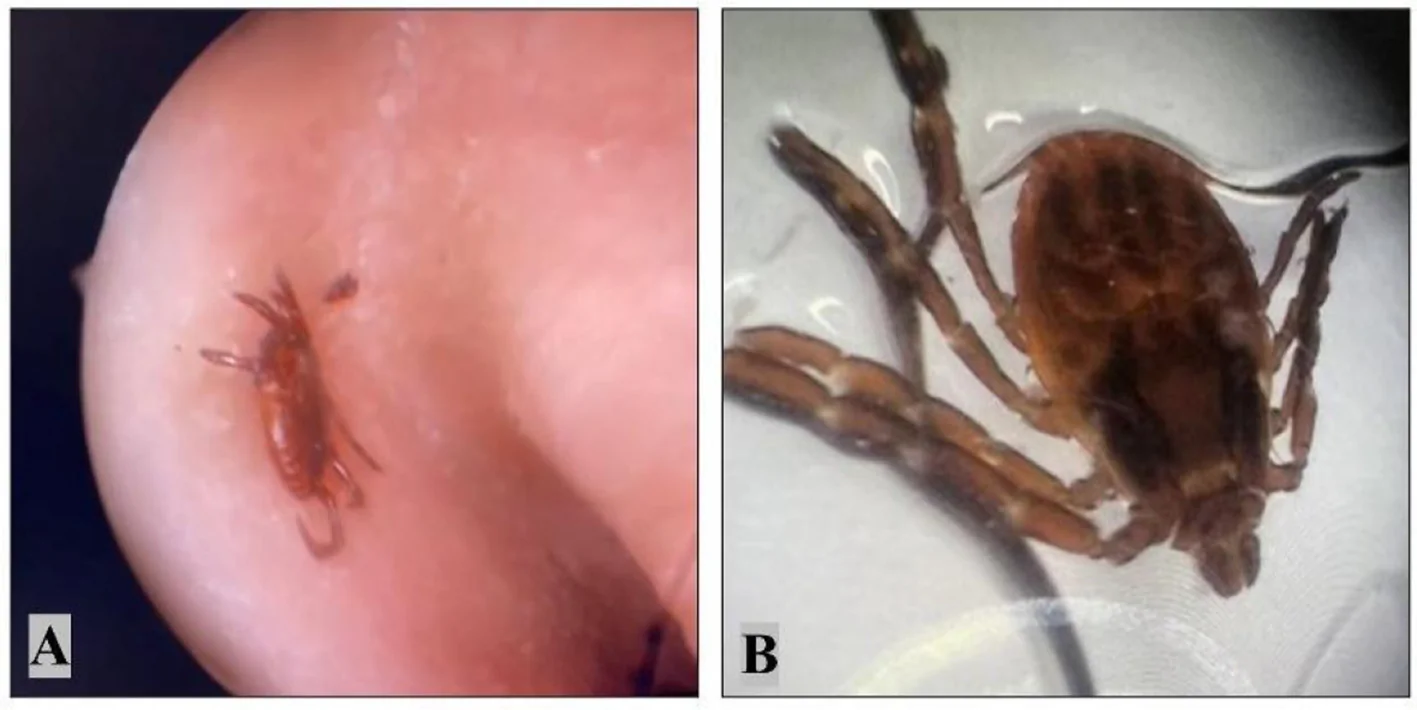
**(A)** An adult tick attached to the wall of the external auditory canal. Under low magnification, the tick appears small and slender, potentially leading to misidentification. **(B)** High-magnification view of the extracted specimen, showing the fully sclerotized scutum and robust legs characteristic of an adult hard tick (family Ixodidae).

## Discussion

Ticks are temporary ectoparasites that feed on vertebrate hosts and serve as vectors for numerous zoonotic pathogens. The three cases presented here, occurring in July 2021, July 2024, and September 2025 in three distinct East Chinese cities (Qingdao, Jinan, and Shanghai), coincide with peak tick activity periods. Crucially, this case series from geographically diverse locations illustrates the One Health paradigm, wherein human health is interconnected with animal reservoirs and environmental factors. It provides evidence that otoacariasis is not an isolated clinical rarity but a recurring issue in East China, spanning both rural and urban settings. This pattern signifies a potential gap in public health surveillance and primary care education, making it a relevant concern for the region's public health infrastructure. This concern is further supported by recent evidence from China. A 2024 study analyzing online search behavior found a mismatch between public concern and actual tick-borne disease risk, with high-risk areas often receiving lower public attention (8). This discrepancy highlights the urgent need for targeted public health education campaigns, particularly in regions like East China where otoacariasis may be underrecognized.

In East China, common tick species include *Haemaphysalis longicornis* and *Dermacentor sinicus* (9), which are known vectors of severe fever with thrombocytopenia syndrome virus (SFTSV) and spotted fever group rickettsiae, respectively. A comprehensive review has documented the diversity and distribution of tick-borne pathogens in China, underscoring their public health relevance(10). Among these, *H. longicornis* is widely distributed in East China and has been implicated in human otoacariasis cases (11), while *D. sinicus* is frequently associated with rural and suburban environments where tick exposure occurs. Ticks typically attach to thin, soft skin areas less prone to disturbance (5, 6), making bites in the external auditory canal rare. Previous reports of otoacariasis have documented symptoms including tinnitus, otalgia, aural fullness, hearing loss, pruritus, otorrhea, and vertigo (11, 12), with rare cases of tick-induced facial palsy (7). Tick bites are often painless due to anesthetic and anticoagulant enzymes in tick saliva, which may delay detection (11–13). Local reactions include pruritus, pain, erythema, and lymphadenopathy, while systemic manifestations can range from fever and headache to tick paralysis, a potentially fatal ascending paralysis (12, 13). Because the risk of complications increases with attachment duration, prompt removal is critical (13).

We highlight diagnostic features of ticks in the ear—morphology and dark red feces (2–6, 11–15)—to aid differentiation from benign conditions. Our three cases illustrate a spectrum of diagnostic and management challenges: an engorged tick abdomen mistaken for the tympanic membrane (Case 1), retained mouthparts after forceful removal (Case 2), and a tick on the tympanic membrane dismissed as a common insect under low magnification (Case 3). Accurate species identification by entomology specialists enabled assessment of tick-borne disease risk (e.g., SFTS, Lyme disease) and guided prophylactic decisions. However, we acknowledge that morphological identification in this case series was limited to the family level (Ixodidae) or probable genus (*Haemaphysalis* or *Dermacentor*), as molecular methods (e.g., PCR sequencing) were not available for these retrospective specimens. Future prospective studies should incorporate molecular identification to confirm tick species and refine risk assessment. We recommend incorporating such identification protocols into otolaryngologic practice, particularly in regions with high tick activity. Given that otoacariasis is rarely reported in East China, these three cases from Qingdao, Jinan, and Shanghai underscore the need for heightened clinical vigilance across the region.

Proper removal is critical to avoid retained mouthparts and secondary infection. In Cases 1 and 3, instillation of 2% lidocaine immobilized the tick, allowing atraumatic extraction with microforceps. In contrast, Case 2 involved forceful removal at a primary hospital, resulting in retained mouthparts requiring surgical excision. These experiences support the use of topical anesthetics before removal to minimize retained fragments (11, 12). Alternative strategies include waiting for spontaneous detachment (11), grasping the tick with curved forceps (15), or excising the abdomen after aspiration (12).

Prophylactic antibiotics remain controversial. The IDSA/AAN/ACR guidelines recommend a single dose of doxycycline for high-risk bites (16), whereas the ILADS guidelines advocate for prolonged doxycycline (100–200 mg twice daily for 20 days) (17). In our cases, prophylaxis was individualized based on the specific tick-borne disease of greatest concern for each patient. In Case 1, an engorged tick was removed from a 70-year-old woman with rural exposure; the main concerns were Lyme disease and tick-borne rickettsiosis, and minocycline was prescribed considering her age, tolerability, and local formulary availability. In Case 2, a 3-year-old girl had retained tick mouthparts after forceful removal; the primary concern was local secondary infection, with lower risk of systemic tick-borne disease because the tick body was absent, and antibiotics were withheld due to her age. In Case 3, a 5-year-old girl had a tick attached to the tympanic membrane, which was completely removed; the main concerns were severe fever with thrombocytopenia syndrome (SFTS) and Lyme disease, but prophylaxis was withheld because removal was complete and the patient was a child. Prophylaxis decisions should consider tick species, attachment duration, and regional disease prevalence.

## Methods

### Study design and participants

This was a multicenter retrospective case series conducted at three tertiary hospitals in East China: the Affiliated Hospital of Qingdao University (Qingdao), Shandong Provincial Hospital Affiliated to Shandong First Medical University (Jinan), and Eye & ENT Hospital of Fudan University (Shanghai). The study period spanned from July 2021 to September 2025. The multicenter design was chosen to capture a broader geographical representation of otoacariasis cases in East China, thereby informing regional public health strategies. Cases were identified by searching electronic medical records using keywords including “tick,” “otoacariasis,” “external auditory canal foreign body,” and “ear foreign body.” Inclusion criteria were a clinical and/or pathological diagnosis of tick bite in the external auditory canal and availability of complete medical records.

Clinical data were extracted from electronic medical records using a standardized form, including demographics, exposure history, clinical presentation, prior misdiagnosis, treatment history, tick identification, and follow-up outcomes. Two investigators extracted and cross-checked the data. All patients underwent endoscopic or otomicroscopic examination. Tick specimens were examined under stereomicroscopy by entomology specialists. Morphological identification was based on the scutum, capitulum, leg segmentation, and mouthpart structures (hypostome and chelicerae). Identification was limited to the family level (Ixodidae) or probable genus (*Haemaphysalis* or *Dermacentor*) because molecular methods were unavailable for these retrospective specimens. Pathological examination was performed for retained mouthparts in Case 2.

Tick removal was performed after instillation of 2% lidocaine into the external auditory canal for 10–15 min, followed by extraction using microforceps. The external auditory canal was irrigated with normal saline to remove residual feces. Post-removal endoscopy confirmed complete removal and an intact tympanic membrane. Prophylactic antibiotic decisions were individualized based on patient age, tick species, attachment duration, and regional disease prevalence.

Patients were followed up at 1 and 6 months (as applicable) via clinical examination and telephone interview. Primary outcomes were complete tick removal without retained mouthparts and symptom resolution; secondary outcomes included tick-borne disease risk based on species identification.

### Outcome measures

Primary outcomes were successful tick removal without retained mouthparts and resolution of symptoms at follow-up. Secondary outcomes included assessment of tick-borne disease risk based on species identification.

## Data Availability

The datasets generated and analyzed in this study are included in the article and its supplementary material (Supplementary Video S1). Raw clinical data are not publicly available due to privacy restrictions but are available from the corresponding author upon reasonable request.
